# Do GPs know more than other doctors? Commentary on: ‘COGITA network has constructed a glossary of diagnostic reasoning terms’

**DOI:** 10.1080/13814788.2017.1282455

**Published:** 2017-03-08

**Authors:** Norbert Donner-Banzhoff

**Affiliations:** ^a^ Department of General Practice/Family Medicine, University of MarburgMarburgGermany

In this issue of the *European Journal of General Practice,* Barais et al., present their glossary of diagnostic reasoning terms [1]. COGITA is a European group of general practitioners (GPs) reflecting on diagnostic reasoning and decision making, perhaps more widely known under their popular name ‘The Gut Feeling Group’. References of their publications can be found on http://www.gutfeelings.eu. They now introduce their definitions of terms related to diagnostic reasoning, such as gut feelings, contextual knowledge, rules of thumb and several more. They have also added terms relevant for research in this area, with construct validity, triangulation or focus group to name but a few. Diagnostic reasoning and decision making have a long tradition, not only with physicians, but also with several other disciplines (cognitive psychology, philosophy, economics, artificial intelligence, et cetera) exploring what goes on in doctors’ heads. Do we really need more of this? Is this a contribution justifying the extra effort?

I suggest that there are two aspects that are typically brought forward by GPs when the diagnostic assessment of patients is discussed explicitly. Both are inextricably linked: one is the acknowledgment of uncertainty in clinical practice and the other is the role of non-analytical methods of reasoning.

One reason to be aware of uncertainty in primary care practice is the kind of problems presented by our patients. Typically, we face unclear, diffuse, undifferentiated problems. If a defined diagnosis comes to mind at all, it is more often an untypical case than a typical. If anything is typical, it is the entanglement of the biological, the psychological and the social.

There are, however, less obvious reasons why GPs are emphasizing what is uncertain in diagnostic reasoning. One is the longitudinal view we have on our patients, which is a major privilege of primary care, especially in healthcare systems where citizens register with a GP who acts as a gatekeeper. While other disciplines use high-tech investigations as the final arbiter of their diagnostic impressions, we have a better one: our patients come back and generously present their long-term outcome to us. This is the most valid kind of hypothesis testing and feedback one can imagine. This experience often gives us a lesson in realism and humility. Perhaps, life is easier for many of our specialist colleagues, who see patients for a single occasion or episode only. If things work out differently from expected after the patient’s discharge from hospital, they often do not learn the truth. This situation may put their minds at ease and boost their confidence, but the feedback they obtain is distorted and thus prevents valid learning [2]. This becomes even more problematic if there is too much reliance on imaging and biochemical tests. Population-based studies have shown the limitations of technical methods in telling us what the patient’s problem really is [3–6].

The other reason why GPs embrace uncertainty easier than other disciplines is the complexity of problems we usually meet in one person. That complex or even bizarre complaints are explained by just one diagnostic term is rare enough. However, even if it happens, the problem is rarely solved that way. There is comorbidity, depressive and anxious reactions, multiple treatments causing side effects and interactions, patients and families have questions about the disease and its treatment, and numerous psychosocial issues.

Therefore, the answer to the question ‘Do GPs know more than other doctors?’ is a clear ‘yes’. However, the added knowledge is more of the Socratic kind, i.e. being aware of the limitations of one’s knowledge and having a realistic view on what medicine can achieve in defining the patient’s problem (diagnosis), making predictions about what is going to happen (prognosis) and improving the patient’s well-being (treatment).

Whenever GPs reason about their decision making, uncertainty is the most persistent theme. In other disciplines, this kind of talk is usually taboo, as is the non-analytical part of making a diagnosis, the rules of thumb, the mind lines, the skilful use of context information (usually ignored in other fields), the gut feelings, heuristics and patterns recognized. All these are essential strategies adapted to the primary care setting where fast decisions must be made with limited time and under conditions of maximal complexity. The open attitude regarding non-analytical strategies gives GPs’ elaborations their specific flavour.

I am convinced that uncertainty and intuitive decision making are as prevalent in other medical fields as they are in primary care. Outside general practice, however, these are carefully kept in the closet. Since primary care is special, since diagnostic reasoning and decision making in primary care is special and related talk is special [7], the glossary by the COGITA group fills an important gap. At present, I can see no other source that could provide this kind of background. However, this can only be a start, there are many more terms waiting to be included. Mind lines, hypothesis testing or deductive reasoning, inductive foraging, error and regret, but also think-aloud-technique, stimulated recall and many more are waiting to be included [8,9]. We hope that this is going to happen soon.
